# Resource Recovery from Wastewater by Biological Technologies: Opportunities, Challenges, and Prospects

**DOI:** 10.3389/fmicb.2016.02106

**Published:** 2017-01-06

**Authors:** Daniel Puyol, Damien J. Batstone, Tim Hülsen, Sergi Astals, Miriam Peces, Jens O. Krömer

**Affiliations:** ^1^Group of Chemical and Environmental Engineering, School of Experimental Sciences and Technology, King Juan Carlos UniversityMostoles, Spain; ^2^Advanced Water Management Centre, University of Queensland, BrisbaneQLD, Australia; ^3^CRC for Water Sensitive Cities, ClaytonVIC, Australia; ^4^Centre for Solid Waste Bioprocessing, School of Civil Engineering, University of Queensland, BrisbaneQLD, Australia; ^5^Centre for Microbial Electrochemical Systems, University of Queensland, BrisbaneQLD, Australia

**Keywords:** circular economy, cradle-to-cradle, resource recovery, water-energy nexus, biological processes, wastewater treatment

## Abstract

Limits in resource availability are driving a change in current societal production systems, changing the focus from residues treatment, such as wastewater treatment, toward resource recovery. Biotechnological processes offer an economic and versatile way to concentrate and transform resources from waste/wastewater into valuable products, which is a prerequisite for the technological development of a cradle-to-cradle bio-based economy. This review identifies emerging technologies that enable resource recovery across the wastewater treatment cycle. As such, bioenergy in the form of biohydrogen (by photo and dark fermentation processes) and biogas (during anaerobic digestion processes) have been classic targets, whereby, direct transformation of lipidic biomass into biodiesel also gained attention. This concept is similar to previous biofuel concepts, but more sustainable, as third generation biofuels and other resources can be produced from waste biomass. The production of high value biopolymers (e.g., for bioplastics manufacturing) from organic acids, hydrogen, and methane is another option for carbon recovery. The recovery of carbon and nutrients can be achieved by organic fertilizer production, or single cell protein generation (depending on the source) which may be utilized as feed, feed additives, next generation fertilizers, or even as probiotics. Additionlly, chemical oxidation-reduction and bioelectrochemical systems can recover inorganics or synthesize organic products beyond the natural microbial metabolism. Anticipating the next generation of wastewater treatment plants driven by biological recovery technologies, this review is focused on the generation and re-synthesis of energetic resources and key resources to be recycled as raw materials in a cradle-to-cradle economy concept.

## Introduction: From Water Remediation to Water Mining: Cradle-To-Cradle in Wastewater

The current societal production system, based on raw matter extraction and industrial transformation into products, has long-term sustainability issues (Lovins, 2008). The main reason is the use of non-renewable inputs, such as fossil fuels, essential agricultural nutrients such as phosphorus and rare metals used in electronic devices production. The need to close production cycles and enable resource sustainability is driving certain regions, especially EU and Japan, to choose for a self-sufficient bio-based economy. These strong drivers are pushing to change current production systems, and the next two decades are key in enabling a sustainable technological society. The circular economy concept anticipates a global sustainable development if the production system becomes auto-regenerative and the waste generated in technical and biological cycles is converted into raw matter. These include agricultural and industrial wastes, as well as those derived from direct human consumption (Pearce and Turner, 1990). This is better known as cradle-to-cradle concept, which is substituting the current and outdated triple-R model (recycle, reuse, and recovery) by a more efficient paradigm where not only the waste is recycled but also is used as raw material, and the whole process is driven by renewable energy (McDonough and Braungart, 2010).

Wastewater treatment is a key platform to base the technological development focused on the change of the production system, since it is worldwide established with a very long technological history (Van Loosdrecht and Brdjanovic, 2014). Between 50 and 100% of lost waste resources are contained in wastewater. Therefore, major drivers, including not only economy and environment expertises but also industrials, are pushing to recover and regain all these substances. The EU has invested substantial resources into bioeconomy and a specific Research and Innovation program was created recently (the Biobased Industries Joint Undertaking^1^, funded by the European Comission under the Horizon 2020 framework). USA was one of the most relevant drivers of the bioeconomy through the National Bioeconomy Blueprint (House, 2012). As President Barack Obama claimed in 2011, “The world is shifting to an innovation economy and nobody does innovation better than America.” These declarations clearly indicated that the USA intends leading the progressive evolution of the global economy toward a new cycle. As will be noted later in this review, this evolution involves also a new generation of wastewater treatment plants, where energy, organics, and other resources are recovered as valuable byproducts instead of being wastefully dissipated or destroyed. This is being driven not only by a need for reduced cost and resource, particularly energy consumption, but is also motivated by worldwide depletion of non-renewable macronutrients such as easliy-accesible phosphorous, and the need to reduce anthropogenic effects on terrestrial nitrogen cycles (Batstone et al., 2015).

While many new technologies are contributing to the challenge of resource recovery from wastewater, biological methods offer the strongest promise to efficiently recover valuable resources from dilute streams. Examples include fast growing heterotrophic, chemotrophic, phototrophic, and photosynthetic bacteria, microalgae, and terrestrial plants for organics recovery, and the use of highly specialized metal reducing and oxidizing organisms for metal recovery. Organisms absorbing complex organics can be used to recover biopolymers such as polyhydroxyalkanoates and alginates can be generated by accumulative bacteria. This review will focus broadly on biological methods to recover resources from domestic and industrial wastewater and industrial wastes. The next generation of domestic wastewater treatment plants (DWWTP) is targeting energy neutrality and complete recovery of nutrients, particularly N and P. There are also increasing drivers to recover valuable products from wastes and wastewaters of different nature, such as those from the industrial manufacturing and mining extraction. These compounds are characterized by their high stability and low biodegradability. Resources that are capable of being recovered by biological technologies includes heavy, precious or radioactive metals, and emerging pollutants like pharmacs, enzymes, hormones, fertilizers, and bioplastics. Despite some efforts have been dedicated to recover these valuable resources, there is still a need for improving and consolidating the biological options to reclaim and reuse these substances.

## Domestic Wastewater as Key Developmental Platform for Nutrient and Energy Recovery

2014 was the 100 years anniversary of the activated sludge process, and has seen commemoration of remarkable advances in human health, standard of living, and improvements in the environment enabled by the activated sludge process over the last 100 years (Jenkins and Wanner, 2014). Each iteration required major investments in infrastructure, with a cycle length of approximately 50 years, which largely aligns with the maximum lifespan of this infrastructure. We are now entering the start of another major cycle, driven partly by the end of life of the current infrastructure, as well as by recognition of a need to reduce global environmental impact and enable long term societal sustainability (Verstraete et al., 2009; Mccarty et al., 2011; Batstone et al., 2015). This aims at reducing the substantial resource consumption (energy, chemicals, and transport) of existing wastewater treatment and enabling instead recovery of the value inherent in wastewater (Daigger, 2009). This part outlines the reasons motivating this, platforms available to enable resource recovery, and the practical application of resource recovery at a small city scale.

Domestic wastewater by itself cannot completely fulfill fertilizer requirements, as there is substantial dissipation to both domestic animal production (not normally captured in urban treatment systems), as well as the environment. Globally, approximately 20% of *manufactured* nitrogen and phosphorous is contained in domestic wastewater (Batstone et al., 2015; Matassa et al., 2015), of which the majority is potentially recoverable due to urban concentration. The situation is more attenuated for energy. Wastewater contains 1.3 MJ/person/day (6.5 MJ/kL) of chemical energy (Batstone et al., 2015). This represents 1% of the current world total energy consumption, or 4% of the world total electricity production (OECD/IEA, 2015), and requires a process to convert it from dilute chemical energy to a usable form. However, it represents a concentrated source of carbon that may be better utilized directly as a resource (Matassa et al., 2015).

Overall, domestic wastewater alone cannot fulfill elemental or energy needs of industrialized society (as the rules of resource dissipation would imply). However, it represents a substantial resource, and should be fully utilized. Finally, the domestic wastewater context has traditionally represented a basis for technology development in waste and wastewater treatment in general, generally due to the increased financial resources available (compared to, for example, agri-industrial waste recovery), and technologies developed in this area are applicable to enable resource recovery across the industrial and agricultural cycle.

There are a number of broad process options to enable lower energy wastewater treatment, including high-footprint passive systems (wetlands, lagoons), low energy mainline anaerobic (e.g., UASBs, Anaerobic MBR), together with alternative nitrogen removal methods such as mainline Anammox (Wett et al., 2013). These are discussed further in this paper. However, most focus only on energy recovery, with dissipation of carbon, nitrogen, and phosphorous and there are a limited number of processes that aim for full or enhanced recovery of these resources.

### Partition-Release-Recover Concept

Verstraete (Verstraete et al., 2009) proposed separation of streams into major and minor (M&m) concentrated and dilute streams. The default sets of technologies identified were filtration based treatment (gravity-microfiltration-reverse osmosis), with treatment of solids and concentrate by anaerobic digestion (AD), and recovery of the nutrients from digestate though [for example, electrodialytic nitrogen recovery (Mondor et al., 2008), and phosphate precipitation]. Verstraete also identified alternatives, including biological concentration through organisms that grow quickly such as heterotrophic activated sludge organisms.

**FIGURE 1:**
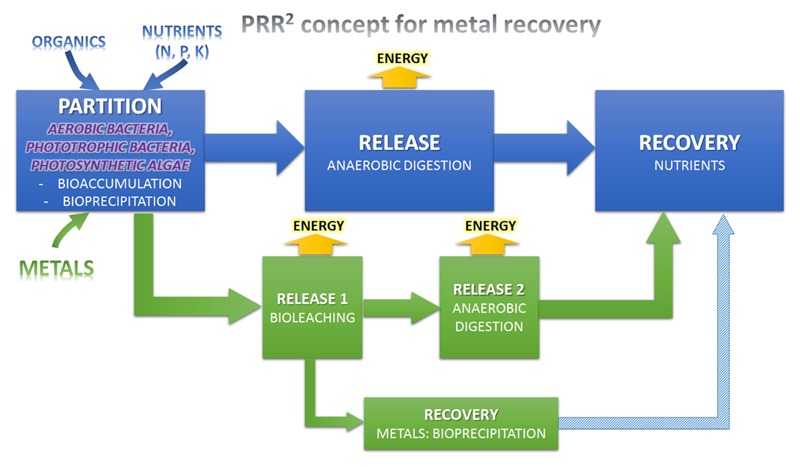
**Enhancing the Partition-Release-Recovery concept for organic and metals recovery from wastewater (PRR^2^ concept)**.

This was further developed, as the “partition-release-recover” process, which uses biological agents to selectively remove nutrients and carbon from the liquid phase (Batstone et al., 2015). This is a combined and scalable process, able to treat wastewater at essentially zero energy input, and recover nitrogen, phosphorous, and potentially, value-added organics or microbial products from the eﬄuent. An overall scheme is shown in **Figure 1**.

This concept has been further developd by (Batstone et al., 2015), and is summarized here. The overall process has a single entry point (wastewater), and four key discharges:

(i) Water, in which the main hydraulic load is dispersed through reusable water, with a defined discharge limit of nitrogen and phorphorous depending on reuse requirements, local regulations, and technology options chosen. This is the main discharge from the “partition” stage. Partial nutrient removal with subsequent treatment may also be affected in the partition stage, with downstream treatment through low energy biological or chemical treatment.
(ii) Biogas, which is the main sink stream for excess chemical energy. This is the energy product from the “release” stage. This is a relatively low value energy stream and an ultimate better goal may be recovering organics as a higher value product (see below).
(iii) Biosolids, mainly composed by inert organics, non-recoverable nutrients and excess metals. This is the byproduct from the “release” stage. It seems to be critical to achieve almost complete AD, otherwise much of the benefits are lost in excess sludge production. However, biosolids can be also used as organic fertilizers if they are fullfil the requirements (Tontti et al., 2016).
(iv) A fertilizer stream, which is the main sink for nitrogen, phosphorous, and possibly potassium. This is the valuable product from the “recover” stage. Again, as commodity chemicals, these have relatively low value, and a better ultimate goal may be generation of valuable products.

The key differentiating feature is the “partition stage,” with a number of different agents available for use. These include:

• Heterotrophic bacteria, where both energy and electron equivalents for growth are chemically sourced from the wastewater (with oxygen as catabolic electron acceptor). This is generally termed high-rate activated sludge, or A-stage treatment, and has been applied for 20 years (Jetten et al., 1997; Jimenez et al., 2015).
• Phototrophic anaerobic bacteria [particularly purple phototrophic bacteria (PPB)], where the energy for growth is sourced from light, but the electrons, carbon and nutrients from the wastewater. This has been demonstrated as a domestic treatment option in the laboratory (Hülsen et al., 2014, 2016b). Technology readiness level (TRL) has to be upgraded before real application of the technology to achieve at least TRL 7.
• Algae and oxygenic photosynthetic bacteria, where the energy for growth and catabolism is sourced from light, electrons from molecular water, and nutrients and carbon (generally as carbon-dioxide). Particularly for heterotrophic treatment, this generally involves participation of aerobic bacteria, which nitrify and oxidize carbon to CO_2_ (Cai et al., 2013). There are some examples of full-scale application of algae processes, though resource recovery is still not fully addressed (e.g., EU FP7 ALL-GAS project, n° ENER/FP7/268208).

Particularly phototrophic is embryonic in nature and algae is still under development, and with limited field application. All three have fundamental restrictions; in particular, energy input and carbon utilization efficiency for heterotrophic bacteria, the need for soluble carbon for phototrophic anaerobes, and light energy and footprint limitations for algae. However, all three enable the generation of value-added products in the form of biomass (and other byproducts) that represent enabling platforms for resource recovery.

### Wastewater Biofactory

The other concept which is emerging is re-engineering of conventional activated sludge, particularly by identifying new byproducts which are far higher value than the raw energy content of the wastewater. Activated sludge naturally concentrates organics in a sludge stream, with partition-release-recover aiming to maximize this. However, a parallel focus is on enhancing conventional activated sludge (Van Loosdrecht and Brdjanovic, 2014), with obvious applicability to other processes.

Enhanced products can be generally split into those that feed into commodity chemical industries, which are purified organic and other chemicals, and composite or complex materials suitable as a bulk input to manufacturing, agriculture, or even consumer use.

Commodity chemical include organic acids and alcohols (including higher molecular weight organics), carbon-dioxide, purified nutrients, and metals. These are discussed further below, but particularly for organics, the two key routes are fermentation and extraction of fermented product (Kleerebezem and Van Loosdrecht, 2007), or recovery of the organics as a concentration, and conversion to syngas for subsequent reformation (Batstone and Virdis, 2014). Both of these are impractical at the very low concentrations available in wastewater, and are better applied on sludge streams.

From a value perspective, it is far better to produce composite products. These include manipulating activated sludge to generate polyhydroxyalkanoates (PHA) (Kleerebezem and Van Loosdrecht, 2007), or a PHA composite, production of long-chain microbial exo-polysaccharides, including alginates (Sam and Dulekgurgen, 2016), particularly through the use of aerobic granular sludge (Lin et al., 2010), and even direct recovery of ubiquitious fibers such as cellulose in wastewater (Van Loosdrecht and Brdjanovic, 2014). These have valuable, or even unique properties. However, production generally utilizes only a fraction of the resource-carbon (or nitrogen) available in the wastewater, and hence should be part of a larger resource recovery strategy.

## Resource Recovery for a Circular Economy

**FIGURE 2:**
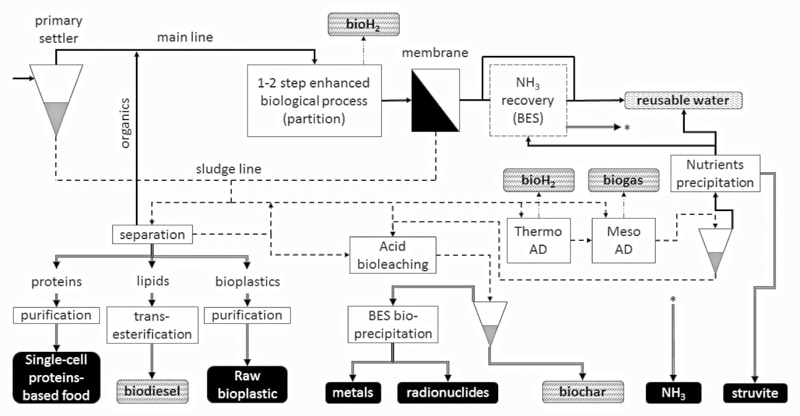
**Conceptual overview of different biological technologies applied in wastewater treatment for energy and resource recovery.** Energetic products are shown as dashed vertical patterned blocks, whereas raw materials are depicted as black blocks. Wide continuous lines are water lines, dash lines are sludge lines, dash-dot-dot lines are gas lines and double lines represents resources production/extraction.

As noted above, there are a broad range of recovery strategies available, with further differentiation based on product. These feed into almost all categories of agri-industry and chemical production, including potentially, energy economy (including vehicle fuels), raw commodity chemicals, manufacturing and composite industrial inputs, fertilizers, animal feeds, other elements, and even consumer products. This section summarizes many of the key products. A schematic representation of common resource and energy recovery lines in a wastewater treatment plant is shown in **Figure 2**.

### Biofuels

The conversion of organic-rich wastewater streams into bioenergy has a long history, especially through AD (Mccarty et al., 2011). Several technologies are under current development to convert organic matter to bioenergy such as biohydrogen, biodiesel, bioethanol, and microbial cell fuels; however, their present feasibilities are far from the reached by anaerobic systems.

#### Biogas

Anaerobic digestion is a commercial technology applied to convert municipal and industrial organic wastewater streams into renewable energy in the form of methane-rich biogas (Batstone and Virdis, 2014). Despite energy recovery, AD present other important advantages such as high organic matter removal efficiency, low excess sludge production and low space requirements (van Lier et al., 2015). Today, AD infrastructure is used to treat a wide variety of organic wastes including (i) sewage sludge, (ii) animal manures, (iii) food and paper industry wastes, including slaughterhouse waste, (iv) energy crops and harvesting residues, including microalgae, and (v) organic fraction of municipal solid waste (MSW) (Romero-Güiza et al., 2016). Nonetheless, digesters configuration is less diverse since most AD plants are either continuous stirred tank reactor or high-rate bed reactors (e.g., upflow anaerobic sludge blanket and expanded granular sludge bed reactors) used for highly particulate and highly soluble wastewater streams, respectively (van Lier et al., 2015; Romero-Güiza et al., 2016). However, the successful and quick development of anaerobic membrane bioreactors (AnMBR) will further expand the application of AD to a range of new substrates within a short period time (e.g., pharmaceutical, municipal sewage, petrochemical, and winery, among others) (Dereli et al., 2012). AnMBR, which combines the advantages of AD and membrane filtration, represent a sound alternative to high-rate bed reactors for intensive AD (Smith et al., 2012). The main advantage of AnMBR over high-rate bed systems is the total retention of particles. Thus, AnMBR (i) produce high quality eﬄuents (free of solids and pathogens) and (ii) retain special microbial communities able to degrade specific pollutants and/or tolerate higher concentration of an inhibitor regardless of its aggregation or sedimentation properties (Dereli et al., 2012). The latter process advantage is key to treat heavy polluted wastewater streams from a variety of industries.

The implementation of AnMBR as a mainline process for domestic wastewater treatment plants appears as a promising technology to improve the economic feasibility of these plants (Smith et al., 2014). The main advantage of this configuration is its capacity to recover most of the energy potential in the wastewater rather than the fraction currently recovered by the aerobic-anaerobic treatment, where the energy potential of soluble organic matter is not recovered but removed by energy-intensive aerobic processes (Mccarty et al., 2011). According to Mccarty et al. (2011) the full anaerobic treatment of municipal sewage by AnMBR will double the energy production of DWWTP, and energy production will exceed the DWWTP energy needs. However, the net energy balance done by Smith et al. (2014) shows that the energy recovery will largely depend on the municipal sewage strength, the membrane flux, and the energy spent on fouling control. Nevertheless, AnMBR feasibility is expected to increase as the technology matures (Mccarty et al., 2011; Dereli et al., 2012; Smith et al., 2014). Beside the energy recovery through biogas production, AnMBR advantages over conventional aerobic systems include lower production of excess sludge and higher eﬄuent quality in terms of solids and pathogens (Dereli et al., 2012; Smith et al., 2014). The latter is especially relevant as reclaimed wastewater reuse (e.g., landscape and crop irrigation and domestic/industrial consumption) becomes a common practice (Mccarty et al., 2011). The main drawback of AnMBR for municipal sewage treatment is that psychrophilic temperature (<20°C) is the only economically feasible option in temperate and cold climates (Smith et al., 2012; Gouveia et al., 2015). The operation of AnMBR at low temperature not only slows down the kinetics of all biological process (organic compounds degradation and biomass growth) but also increases the dissolved methane in the eﬄuent as methane is approximately 1.5 times more soluble at 15°C compared to 35°C (Lettinga et al., 2001; Lin et al., 2013; Ozgun et al., 2013). The recovery of the dissolved methane is key to reach an energy-neutral operation for domestic wastewater but also to reduce greenhouse gas emissions (Smith et al., 2014). Several technologies have been developed to minimize and recover the dissolved methane in the eﬄuent. Giménez et al. (2012) reported that biogas-assisted mixing avoids oversaturation and guarantees a minimum dissolved methane concentration in the AnMBR eﬄuent (the average oversaturation value for AnMBR eﬄuents is 1.5). Stripping the dissolved methane with air seems the simplest option to reduce the methane concentration below the saturation concentration as well as to add oxygen to the eﬄuent stream (Mccarty et al., 2011). The use of degassing membranes has also been suggested (Bandara et al., 2011); nonetheless the energy requirements can be higher than the energy recovered. Another alternative is to use the dissolved methane as carbon source for methanotrophs, which can be combined with other biological process such as denitrification (Strong et al., 2015) and bioplastics production (Strong et al., 2016).

While complete anaerobic treatment of municipal sewage has possibly the highest potential for recovering wastewaters organic energy content, revamping existing aerobic-anaerobic DWWTP to anaerobic facilities could be costly and therefore it may only be possible for new infrastructure (Mccarty et al., 2011).

Another opportunity to reduce the energy needs in DWWTP is anaerobic co-digestion (AcoD) (Mata-Alvarez et al., 2011). AcoD, the simultaneous AD of two or more substrates, is a proven approach to overcome the drawbacks of single digestion, boosting the energy production in AD plants to redirect waste away from landfill toward reuse while utilizing existing infrastructure (Jensen et al., 2014; Mata-Alvarez et al., 2014). AcoD is especially useful for AD systems that are operating under capacity and therefore able to receive onsite or external waste for combined treatment. Although literature values are scarce, up to 30% spare capacity has been reported for DWWTP sewage sludge digesters (Fonoll et al., 2015). MSW and fruit and vegetable waste from food processing industries are the most studied and applied co-substrates in sewage sludge digesters; and several successful full-scale AcoD experience at DWWTP have already been reported (Mata-Alvarez et al., 2014). Zupančič et al. (2008) increased by 45 and 130% the heat and electricity energy, respectively, when increasing the loading rate of the Velenje DWWTP sewage sludge digesters (Slovenia, 50,000 p.e.) a 40% (COD-based) with MSW. Similarly, Koch et al. (2016) reported that the self-generated energy at the DWWTP Garching/Alz (Germany, 30,000 p.e.) increased from 25% up to 78% when adding 10% food waste (mass-based) and substituting the old combustion heat and power units. The authors also estimated that the DWWTP could be self-sufficient if the food dose was increased to 16% (Koch et al., 2016). However, the feasibility of using MSW (even sorted) as co-substrate is dependent on the implementation and operational costs of the MSW conditioning (removal of undesired materials and particle size reduction). In this matter, Krupp et al. (2005) and Bolzonella et al. (2006) reported an industrial cost of 50 and 40 € per ton for MSW co-digestion at the Wiesbaden (Germany, 130.000 p.e.) and Treviso (Italy, 70.000 p.e.) DWWTP, respectively. AcoD between fat, oil and greases (FOG) from the DWWTP grit chamber and sewage sludge have also been trialed in several DWWTP (Long et al., 2012). The use of this onsite waste not only allows improving the performance of the digesters but also represents saving the cost of treating the residue outside the plant. However, FOG dosage is limited to an extra loading rate of 1.0–1.5 kgVS m^-3^ d^-1^ due to long chain fatty acids inhibition (Mata-Alvarez et al., 2014).

It is evident that the need to make DWWTP energy-neutral is making AcoD an emerging practice; in fact, it is likely that most medium to large size DWWTP will shortly practice AcoD (Arnell et al., 2016). Despite higher biogas yields, AcoD implementation has an impact on DWWTP performance such as supernatant nutrient content, sludge dewaterability, biosolid quality, and biogas composition (i.a. H_2_S); all of them directly impacting the DWWTP economic balance. In this sense, Arnell et al. (2016), who modeled AcoD using the Benchmark Simulation Model no. 2, observed that while AcoD had a positive effect on the methane production it negatively affected the eﬄuent water quality and the aeration indexes as well as increased sludge production. Therefore, co-substrate selection and dose should be carefully evaluated since random or heuristic decisions on the co-substrate proportion can negatively affect DWWTP performance (Mata-Alvarez et al., 2011).

#### Biohydrogen

Hydrogen (H_2_) has emerged as a valuable energy carrier since it does not produce CO_2_ during combustion, and it has a high energy density per unit mass (Roy and Das, 2015). The dominant technologies for H_2_ production use fossil fuels, consume a lot of energy and have a high carbon footprint; these include natural gas steam reforming (50% world’s production), oil reforming (30%), and coal gasification (18%) (Dincer and Acar, 2015; Roy and Das, 2015). Sustainable hydrogen production needs to rely on environmentally friendly and cost-effective technologies (Dincer and Acar, 2015; Kumar et al., 2015). Biological processes, both autotrophic (e.g., biophotolysis) and heterotrophic [e.g., photo-fermentation and dark fermentation (DF)], are among the more environmentally benign methods for H_2_ production (Das and Veziroglu, 2008; Dincer and Acar, 2015). However, DF is the only technology that accomplishes the dual goal of waste treatment and energy recovery as it utilizes organic waste and wastewater as feedstock (Han and Shin, 2004; Das and Veziroglu, 2008).

Dark fermentation is a process (part of the full AD process) where anaerobic and facultative bacteria degrade carbohydrate-rich substrates into simpler organic compounds [mainly volatile fatty acids (VFA)] with simultaneous production of H_2_ (Turon et al., 2016). DF feasibility is largely limited by its low hydrogen yield, maximally 4 mol of H_2_ per mol of glucose (i.e., it can only recover up to 33% of the biomass energy content). Nonetheless, literature values using mixed cultures and real waste streams (e.g., food waste, lignocellulosic feedstock, agro-industrial waste) rarely exceed 2 mol of H_2_ per mol of glucose (Ghimire et al., 2015; Zhang et al., 2016). Besides the low H_2_ yields, DF commercial feasibility is also limited by several other factors, including (i) controlling the process end product and H_2_ yield, (ii) reducing the presence of H_2_-consumers microorganisms such as methanogens and homoacetogens, and (iii) using DF “biogas” as combustible in hydrogen fuel cells (Levin and Chahine, 2010; Ghimire et al., 2015; Roy and Das, 2015). Furthermore, poorly biodegradable substrates such as waste activated sludge and lignocellulosic residues need to be pre-treated (e.g., ultrasonic, acid, alkaline, and thermal) to reach acceptable H_2_ yields (Noike and Mizuno, 2000; Cai et al., 2004; Levin et al., 2006; Panagiotopoulos et al., 2009).

It does not seem likely that DF will displace medium-term AD as the main technology to convert organic-rich waste and wastewater streams into bioenergy. However, the capability of DF to procure an eﬄuent rich in VFAs (mainly acetate and butyrate) makes it very attractive to be combined with other process for energy and chemical production as well as to further stabilize the DF eﬄuent. Biogenic process that can be combined with DF comprise photofermentation (H_2_ production), microbial electrolysis cells (H_2_ production), AD (methane production), microalgae cultivation (add-value products production), bioplastics (PHA production), and sulfate reduction to H_2_S (metals precipitation) (Muyzer and Stams, 2008; Ghimire et al., 2015; Roy and Das, 2015; Turon et al., 2016). Compared to undeveloped alternatives, the combination of DF and AD in a two-phase anaerobic system represents a plausible modification for existent AD infrastructure (Cavinato et al., 2011; Giuliano et al., 2014). The DF followed by AD process (also known as biohythane) produces a H_2_-rich biogas which improves the thermal efficiency and power output as well as reduces the pollutant emissions of the combustion engine (Porpatham et al., 2007; Moreno et al., 2012). Although the higher efficiency of the two-stage AD over the traditional one-stage AD configuration has been largely proven (Liu et al., 2006; Ge et al., 2010; Riau et al., 2012; Schievano et al., 2014), its full-scale implementation only represents a small fraction of the existing AD infrastructure for municipal waste and wastewater treatment. For instance, in Europe, two-phase systems only represent 7% of the AD infrastructure treating MSW (∼250 plants) (De Baere and Mattheeuws, 2015). According to De Baere and Mattheeuws (2000, 2010), the advantages of the two-stage systems are not enough to compensate the higher investment cost and operation complexity. On the other hand, a multi-stage system (two or more reactors) is the most applied configuration in Germany for the treatment of animal manures, energy crops and other co-substrates; however, the treatment conditions are set to maximize the methane recovery and mitigate emissions rather than producing H_2_ (Weiland, 2006; Lebuhn et al., 2014).

#### Biodiesel

Biodiesel is a carbon-neutral energy source to partly replace fossil fuels, especially in the transport sector which is responsible of 23% of the world’s greenhouse gasses emissions (Muniraj et al., 2015; Zhang et al., 2016). Although H_2_ and methane (after biogas upgrading) can also be used as vehicle fuels, biodiesel represents a smoother alternative since it can be used in existing engines as well as distribution and supply infrastructure without major modifications (Yusuf et al., 2011; Zhang et al., 2016). Today, most biodiesel (>95%) is produced from the transesterification of edible vegetable oil (e.g., canola, palm, rapessed, and soybean); the so-called first-generation biofuels. However, due to the associated food-versus-fuel competition for land and water the production of biodiesel from non-edible oils (second-generation biofuels) is gaining attention (Ashraful et al., 2014; Jin et al., 2015).

Oleaginous microorganisms (microbes able to accumulate more than 20% of their dry weigh as oil) including microalgae, fungi, yeast, and bacteria, are a promising alternative to vegetable oil since they have faster growth rates than plants (Jin et al., 2015; Muniraj et al., 2015). Feedstock cost is one of the main challenges to make microbial biodiesel profitable; therefore, the combination of microbial lipids production and waste and wastewater treatment has been carefully examined (Ashraful et al., 2014; Jin et al., 2015; Muniraj et al., 2015). Among them, the combination of municipal wastewater treatment and microalgae-based biofuels by phototrophic microalgae in high rate ponds algal has been largely investigated (Craggs et al., 2011; Park et al., 2011; Pittman et al., 2011; Mehrabadi et al., 2015). However, this approach is still limited by several drawbacks linked to microalgae growth rates, lipids yield, and lipid extraction. While lipid extraction challenges are shared among all oleaginous microorganisms (Milledge and Heaven, 2014; Jin et al., 2015), limitations affecting phototrophic microalgae growth rates and lipid yield include (i) light penetration and cells mutual shading, (ii) the supply of nutrients and CO_2_, (iii) avoiding contamination by microbes and toxic compounds from the wastewater, and (iv) achieve high lipid yields at high growing rates rather than at under stress treatment conditions (Scott et al., 2010; Liang, 2013; Ward et al., 2014; Mehrabadi et al., 2015). Heterotrophic microalgae can overcome most of the limitations linked to phototrophic microalgae cultivation together with faster growing rates and higher lipid yield (Miao and Wu, 2006; Lowrey et al., 2015). Nonetheless, the dual goal of municipal sewage treatment and lipid production is currently unfeasible (Liang, 2013). Heterotrophic microalgae have also been successfully cultivated using several waste and wastewater streams including molasses (cane, sorghum), crude glycerol (by-product of biodiesel production), sugars from lignocellulosic feedstocks, and VFA-rich eﬄuents from DF (Liang, 2013; Turon et al., 2016). The combination of bioH_2_ production and biodiesel by heterotrophic microalgae seems an interesting approach to achieve waste treatment and energy recovery. However, the main challenges are tuning DF (pH, HRT, and temperature) toward a desired VFA distribution and concentration, as well as avoiding bacterial contamination (Turon et al., 2016). Finally, it is worth highlighting that fungi, yeast, and bacteria are emerging as microbial oil producers. Although this approach is still on its early stages their high growth rates, productivities and yields while using a wide variety of carbon source makes them a worth considering alternative to microalgae-base microbial lipids (Muniraj et al., 2015; Whiffin et al., 2016; Zhang et al., 2016).

### Biopolymers

Polyhydroxyalkanoates (PHA) are biologically produced polymers with similar thermomechanical properties than petrochemical derived plastics such as polyethylene and polypropylene (Laycock et al., 2013). The most common PHAs are homopolymers of 3-polyhydroxybutyrate (PHB) and copolymers with 3-hydroxyvalerate (HV). Other PHA monomers comprise, 3-hydroxybutyrate, 3-hydroxy-2-methylbutyrate, 3-hydroxyvalerate, and 3-hydroxy-2-methylvalerate, and 3-hydroxyhexanoate (Pisco et al., 2009). PHA is a widespread microbial mechanism in nature to store carbon and energy within the cells under unfavorable growth and nutrient conditions as insoluble corpuscles in the cytoplasm. The metabolism of PHA synthesis is encoded by *phaC* gene, characteristic of microbes with PHA-storing capacity.

Although PHA production is a well-known process, its production as a bioplastic commodity is hindered by: (i) the use of pure cultures and sterile feedstocks, which contribute to the high costs production, (ii) PHA yields from mixed cultures, and (iii) extraction and purification methods (Fernandez-Dacosta et al., 2015). However, due to the considerable interest in the emerging bioeconomy many waste streams and low-value feedstocks are suitable targets for bioplastic production, including municipal wastewater and sludge, and agro-industrial wastewaters (e.g., molasses, paper mill, oil mill, and dairy), and spent glycerol. The use of waste streams in combination with mixed microbial cultures have increased the feasibility and sustainability of the biopolymers since the costs associated with the feedstocks are decreased, and the operation and maintenance of the process is simplified.

Currently, the most common configuration of the PHA production process is a three-step process, where different reactor configurations and microbes are involved. The first step is the pre-fermentation of the waste stream, where hydrolytic and fermentative bacteria break down complex organics to readily biodegradable compounds, such as VFA. The second step is the enrichment, where activated sludge is the most common seed biomass. The previously produced VFA are used under dynamic feeding strategies (e.g., by imposing feast/famine and presence/absence of electron donor) to enrich the seed sludge with microbes that possess a high PHA storing capacity. Finally, the third step consists in the PHA accumulation in batch systems, where the biopolymer content of the previously enriched community is maximized (Albuquerque et al., 2013; Moralejo-Garate et al., 2014).

In the pre-fermentation step, the complex waste streams are fermented to obtain VFA, precursor chemicals that can be used as easily and readably carbon source for PHA production, increasing PHA accumulation yields. Although the direct use of non-fermented streams is possible, complex substrates may not be completely degraded, therefore decreasing accumulation yields. Moreover, it has been found that complex substrates can promote the growth of non PHA-storing biomass (Albuquerque et al., 2011; Basset et al., 2016). Although waste pre-fermentation can be seen as an extra step that can increase the costs it can also generate opportunities in a circular economy concept. As shown in (Peces et al., 2016) primary sludge can be inexpensively pre-fermented in an open tank at 20°C obtaining a rich stream in VFA and increasing the methane yield of the remaining solid fraction, improving the feasibility of the process.

The enrichment step is crucial to select microorganisms with high PHA storing capacity and decrease the non PHA-storing populations. The enrichment step has shown to improve storing PHA yields from 4 to 40–64% (% PHA by dry cell) after 50 days of enrichment via aerobic dynamic feeding (Lee et al., 2015), where the enrichment procedure depends on the (i) cycle length, (ii) absence and presence of carbon source in an aerobic system, and (iii) alternating absence and presence of an electron acceptor (aereation). Despite the widespread ability of microbes to store PHA, some of the PHA-storing bacteria found after enrichment belong to genera and species *Amaricoccus* (Lemos et al., 2008) *Azoarcus, Thauera, Paracoccus* (Albuquerque et al., 2013; Carvalho et al., 2014) *Acidovorax, Zooglea* sp. (*Rhodocycales), Hydrogenophaga*. *Rhodococcus* (Morgan-Sagastume et al., 2015) *Flavisolibacter* (Janarthanan et al., 2016), *Lampropedia hyalina* (Villano et al., 2010b).

In the accumulation step, the enriched biomass is transferred into batch-fed reactors, where the final yields will depend on several factors (i) substrate type, (ii) nitrogen (ammonia) limitation, (iii) pH, (iv) organic loading rate, and (v) accumulation cycle length (Johnson et al., 2010; Chen et al., 2013; Pittmann and Steinmetz, 2014). Jiang et al. (2011a) obtained up to a 90% of PHA by dry cell content when using activated sludge enriched in SBR with lactate as substrate, by *Plasticicumulans acidivorans* a novel gammaproteobacterium, which nearest relatives are found to the genera *Methylocaldu*m (Jiang et al., 2011b). However, the monomeric distribution of PHA will directly influence the bioplastic properties, therefore not only is desirable to have high PHA yields but also a stable and robust biopolymer composition. The biopolymer composition is known to be dependent of the VFA distribution of the feedstock during the accumulation (Laycock et al., 2013), where propionate has been correlated with the higher HV percentages (Serafim et al., 2008); but also could be dependent on the metabolic pathways used of the different PHA-storing biomass. Recently, Janarthanan et al. (2016) found that the fluctuations in the microbial population of an enriched culture using pre-fermented whey permeate were not correlated with the final biopolymer composition but with the ratio of acetate-to-propionate in the substrate feed, suggesting mixed culture PHA production as a functionally robust process. In contrast, Carvalho et al. (2014) found that the dominant genera of enriched in PHA-storing microrganisms was *Paraccocus*, the final biopolymer consisted on a 13% of HV, while communites dominated by *Tahurea/Azoarcus* the HV content was consistely higher (20%). Nevertheless, there is still no consensus in the literature to what extent microbial population shifts influence in PHA final distribution.

The PHA production can be combined with other wastewater treatment plant processes such as organic and nutrients removal. For instance, Morgan-Sagastume et al. (2015) successfully integrated the PHA-storing enrichment step with the treatment of the readily biodegradable COD from influent wastewater, with average COD removals of 70% in a pilot scale feast/famine SBR. The enriched PHA-storing community was transferred to the PHA accumulation reactor, where it was fed with VFA-rich stream from waste activated sludge pre-fermentation obtaining PHA productivities of 38% PHA by dry cell. Within the same concept, Basset et al. (2016) presented a novel aerobic feast and anoxic famine enrichment process with a short-cut SBR, where nitritation/denitritation takes place with the simultaneous selection of PHA storing biomass. The authors removed 83% of the nitrogen and the biomass had an 11% PHA by dry cell.

The integration of different operational units within the treatment plant allows to improve the nutrient and energy recovery from the wastewater. A less developed alternative is the use of methanotrophic bacteria to convert C1 compounds, as methane, into PHA. Methanotrophs are mainly a subgroup of gamma and alpha proteobacteria, which are present in several natural environments oxidizing methane to carbon dioxide in presence of oxygen (Strong et al., 2016). However, some methanotrophic bacteria have been found to produce the poly-3-hydroxybutyrate (PHB) homopolymer from methane under nutrient limited condition. This ability has drawn researchers’ interest to use methane as an alternative source to produce value-added compounds (Karthikeyan et al., 2014; Strong et al., 2016). Up to 67% of PHB can be theoretically produced (Asenjo and Suk, 1986). PHB yields using pure methanotrophic cultures, such us *Methylobacterium organophilum* or *Methylocystis* sp., range between 28 and 57% by dry cell depending on the accumulation conditions used (i.e., nutrient limitation conditions) (Weiland, 2006; Zuniga et al., 2011). The use of mixed cultures has focused mainly on synthetic mixtures of methanotrophs under aseptic conditions, with PHB yields of 25% by dry cell under nitrogen limiting conditions (Pieja et al., 2012) and 33% under potassium limiting conditions (Helm et al., 2008). However, the integration of PHA production from methane in a circular economy requires further development on (i) the use of methanotrophic mixed cultures from true natural sources, (ii) accessible enrichment procedures, and (iii) the improvement growth yields due to methane and oxygen mass transfer limitation. Recently, Myung et al. (2015) has enriched a methanotrophic mixed culture for PHA production using activated sludge as inoculum source. The enrichment was carried out in 160 mL serum bottles using a feed-batch strategy where the population was dominated by *Methylocystis.* The enriched biomass was able to accumulate a 39% of PHB by dry cell under nitrogen limitation. The improvement of mass transfer limitation could be optimize by the use of high-rate reactor configurations (i.e., pack-bed columns, fluidised bed reactors). By way of example, Pfluger et al. (2011) enriched a methanotrophic community from a hot spring sediment for PHB production in a fluidized bed reactor, resulting in high-density biofilms achieving up to 20–40% of PHB by dry cell under nitrogen absence. Finally, an unexplored alternative to decrease the methane mass transfer limitation could be to integrate the eﬄuent of AnMBR, rich in dissolved methane using high-rate reactors, to enrich and accumulate PHA from methanotrophs.

### Single Cell Protein

Single cell protein (SCP), referring to edible microorganisms with high protein content, has been applied on an industrial scale since 1919 but after several breakthroughs and intensification on mass SCP production, e.g., baker’s yeast, advances in plant breeding and agriculture after the 1950s simply outcompeted SCPs based on lower costs (Ugalde and Castrillo, 2002). Plant protein was abundant and cheaply available. Today, the food supply for an estimated 7.0 billion people is associated with growing demand for limited resources. Protein scarcity especially in third world countries is a major problem and the demand and costs of conventional protein sources for human and animal consumption are increasing, leaving more than a billion undernourished people (FAO, 2009).

At the same time, the current conversion of fertilizer-nitrogen into edible plant protein is subjected to inherent losses with only 30% of the nitrogen ending up in the plant due to dissipation via run-off and volatilisation (Matassa et al., 2015). Plant breeding is the basis of the food chain all over the world and currently between 75 and 80% of agricultural land is used to grow plants to feed livestock (Foley et al., 2011; Cassidy et al., 2013). However, transforming plant protein into animal protein adds additional conversion losses (Flachowsky and Meyer, 2015). In total, only around 17% of the total fertilizer-nitrogen is retained in vegetable and meat protein with the rest being dissipated (Bodirsky et al., 2014; Matassa et al., 2015). A more efficient use of fertilizer-nitrogen but also a more efficient recovery of nitrogen from waste sources, e.g., via microbial resynthesis has the potential to enable a biobased circular economy (Matassa et al., 2015). Used nitrogen can be recovered and harvested as microbial protein from waste streams (close to 100% recovery) (Shi et al., 2007; Hülsen et al., 2016b) and used directly as organic fertilizer or food for animals (Kobayashi and Tchan, 1973) as well as humans (Becker, 2007). This would at least partly rectify the current inefficiencies whereby the revival of SCP promises alternative proteinaceous food and fertilizer sources for the future.

#### Alternative Sources for Protein Production

Single cell protein can be produced by microalgae, fungi, and bacteria and can be used to substitute for conventional agricultural products such as fishmeal and soy, which are major components in feed formula for aquaculture and livestock (Van Huis et al., 2013). Various sources of SCP were trialed as feed additives for cattle, sheep, swine, poultry, and fish (Hintz et al., 1966). The main advantages of microorganisms for protein production are rapid growth, high protein content and the ability to grow on a wide range of substrates (Tusé and Miller, 1984). These alternatives are less dependent (or not at all) of climate, weather, soil characteristics and available land (Moraine et al., 1979).

However, the applicability of SCP as feed additive depends on the composition. Most microalgae can be feed directly to, e.g., cattle but the algal cell walls need to be digested when feed to monogastric organisms (FAO, 2010). Although microalgae are generally less likely to produce toxins and significant amounts of different species are used for animal as well as human nutrition (e.g., *Spirulina, Scenedesmus, Chlorella, Dunaliella*) (Becker, 2004), the risk of contamination with toxin producing species can only be excluded in pure cultures whereby mixed culture, especially in open systems, have to be closely monitored. Problems might arise from certain species of cyanobacteria which can be part of the consortium and are known to produce toxins (Aráoz et al., 2010).

Single cell protein from fungal biomass, specifically yeast, has been used for a century as animal and human feed additive (Ugalde and Castrillo, 2002). However, depending on the species, fungal SCP might contain elevated levels of nucleic acids and mycotoxin which have to be removed prior of application as feed additive. Both are known to cause severe health effects in humans as well as animals (Speijers and Speijers, 2004). The same applies to bacterial biomass. Depending on the species, a whole variety of endo- and exotoxins can be produced (Anupama and Ravindra, 2000) and the nucleic acid content is generally high (up to 16% of dry weight) which limits the daily intake to a couple of grams per day (Hedenskog and Ebbinghaus, 1972). PPB (Shipman et al., 1975) generally do not produce toxins and have nucleic acids contents comparable to algal biomass and seem suitable as feed additive (Ponsano et al., 2004). A summary of the SCP composition of the different types is given in **Table 1**.

**Table 1:** Composition of Single cell protein (SCP) from the different sources in % of dry weight.

Component	Microalgae	Fungi	Bacteria	PPB
Total nitrogen (Protein + nucleic acids)	45–65^a^	35–50^a^	60–80^a^	60–65^a^
Fats/lipids	5–10^a^	2.6–13^a^	8–10^a^	0.5–9.9^a^
Carbohydrates	9.0	NA	10	21–26^a^
Bile pigment and chlorophyll	6.0	NA	NA	1.7–2.8
Nucleic acids	4.0–6.0^a^	3.9–9.7^a^	15–16^a^	4.3–5.9^a^
Mineral salts	7.0	6.6	8.6	NA
Amino acids	NA	54	65	38.6^b^
Energy content (MJ kdDS^-1^)^c^	10.9–16.1	7.7–14.1	14.7–18.8	13.7–18.9

*Table adopted from (Anupama and Ravindra, 2000). Additional sources: (Shipman et al., 1975; Ivarson and Morita, 1982; Blankenship et al., 1995; Ponsano et al., 2004; Gao et al., 2007), NA- Not available, ^a^depends on the substrate, ^b^only specific amino acids, ^c^alculated based on 16.7, 16.7, and 37.7 MJ kg^-1^ for carbohydrates, proteins and fats as per (Angelidaki and Sanders, 2004).*

Besides the general biomass characterisation, the essential amino acid content, required for animal and human nutrition, plays an important role in the SCP evaluation. **Table 2** shows the essential amino acid content of the relevant SCP source in perspective to egg albumin, a well-balanced reference source for human nutrition. The table shows that the amino acid profiles of each SCP source compare well with egg albumin whereby microalgae and fungi are deficient in sulfur containing amino acids, specifically cysteine. The data of cysteine are missing for PPBs.

**Table 2:** Essential amino acid composition of the different SCP sources and egg white as reference as weight percentage of total amino acids.

Amino acids	Egg^c^	Microalgae^a^	Fungi^b^	PPB^c^
Lysine	5.5–6.1	5.1–6.3	4.1–8.5	5.6–6.0
Threonine	2.9–4.3	4.0–5.9	2.2–3.4	2.9–4.3
Methionine	3.0	1.4–3.2	0.4–1.4	3.0
Cysteine	2.4^b^	0.38–0.65	0.9^b^	NA
Tryptophan	1.6^b^	0.86–1.6	Trace	NA
Isoleucine	3.1–4.3	3.4–5.8	0.5–3.8	3.1–4.3
Leucine	7.4–7.9	7.2–9.0	1.4–4.9	7.5–7.9
Valine	6.5–7.0	5.7–6.7	1.2–5.4	6.4–7.0
Phenylalanine	4.3–4.6	5.4–7.1	0.6–2.3	4.4–4.6

*Sources: ^a^*Bacillariophyceae, Chlorophyceae*, and *Cryptophyceae* (Brown, 1991); ^b^*A. niger, F. oxysporum, F. moniliforme, C. tropicalis* (Christias et al., 1975); ^c^*Rps. Gelatinosa* (Shipman et al., 1975).*

#### Feasibility

After agricultural products outcompeted SCP, the cost of SCP today are still higher compared to conventional products and production costs cannot fully be covered by the product itself (Ugalde and Castrillo, 2002). Although resource limitation is expected to change this picture in the future, the overall SCP production costs have to be reduced. SCP production can be combined with liquid and/or solid waste treatment. As such, algal ponds can effectively treat wastewater and removed constituents are utilized for algal SCP production (Zittelli et al., 2013). The same has been reported for PPB whereby considerable less research has focussed on this source (Hülsen et al., 2014). In this context, the production of fungal SCP from lignocellulose with solid state fermentation, e.g., white rot fungi does not seems to be profitable, mainly due to transport and fermentation costs (FAO, 2010). However, when the raw materials are low-cost and optimum culture conditions are achieved liquid fermentation and SCP from fungal biomass seems feasible (Begea et al., 2012).

Single cell protein can be additionally used as probiotics. Thousands of tons of microalgae, fungi, and bacteria are used as mixed probiotics for aquaculture worldwide every year, particularly in China [up to 50,000 ton per year for (Qi et al., 2009)]. However, probiotic additions to, e.g., fish ponds are not necessarily considered as SCP feed additives because probiotics are added to the water (with other natural occurring bacteria) rather than being the feed. However, the market for probiotics is massive (globally 19,600 million USD in 2013) and several products are commercially marketed but the production occurs mainly batchwise due to difficulties of industrial scale up (Martínez Cruz et al., 2012).

#### Wastewater Treatment and Single Cell Protein Production

Large-scale installations are required to resynthesise protein from wastewater constituent in reasonable amounts. For microalgae the protein resynthesis depends on the wastewater, the loads and residence time but also the nitrogen removal efficiency which was reported to be between 36 and 87% for open high rate algal ponds (HRAP) (Shoener et al., 2014). Areal productivities vary between 1.6 and 23.5 g_biomass_ m^-2^d^-1^(National-Research-Council, 2012) whereby carbon is partly provided from the wastewater but extra CO_2_ is added in most of the cases to drive autotrophic algal growth (Park et al., 2011). Assuming abundant sun energy and carbon supply, nitrogen becomes limiting for the protein synthesis. In this perspective, the main mechanism for nitrogen removal from HRAP is ammonium stripping (Picot et al., 1991; El Halouani et al., 1993) and up to 50% of the nitrogen is dissipated and around 25% are assimilated by the microalgae (García et al., 2000). Additionally, treating non-sterile wastewater is likely to diversify the HRAP community which be comprised of microalgae, plankton, detritus, and terrestrial plant debris, referred to as “Albazod” (Maazouzi et al., 2008) which is likely to impact typical microalgal SCP composition. Since current investment costs exceed the price for economic wastewater treatment with microalgae in closed photo bioreactors by far (Posten, 2009) the SCP production in open systems is limited and losses are only slightly lower compared to agricultural losses [70% loss, (Matassa et al., 2015)].

In this context, the anaerobic growth of PPB as SCP in a closed photobioreactor seems promising as nitrogen removal is non-destructive and losses were reported to be less than 10% with 90% being incorporated into biomass (Hülsen et al., 2016b). Complete assimilation of nutrients by PPB depends on the available organics which have to be added for domestic wastewater but are abundant in industrial sources. Additionally, PPB dominance in a treatment system can be up to 90% (Hülsen et al., 2016a) which would enhance SCP composition. Other bacteria with high nutritional potential such as *Cellulomonas* sp. and *Alcaligenes faecalis* were applied to degrade cellulose containing substrates by solid fermentation of, e.g., wheat straw to enrich proteins (Han, 1975). Complete wastewater treatment systems based on these organisms are not reported in literature although *Cellulomonas* sp. was applied for bio-augmentation in wastewater treatment (Chin et al., 1996).

Also fungal biomass is predominantly applied for solid fermentation whereby wastewater treatment is limited to specific applications such as color and heavy metal removal (Kapoor and Viraraghavan, 1995; Pokhrel and Viraraghavan, 2004). Literature about main line organic and nutrient removal with focus on SCP production is limited (Pant and Adholeya, 2007). Fungal biomass can be applied for sugar and starch containing wastewaters although commercially available technologies would utilize bacteria (anaerobic or aerobic). However, several solid wastes such as corn and sorghum have been used to produce SCP (Anupama and Ravindra, 2000). Other substrates include cellulose and lignin but the pre-treatments to produce accessible substrate is often prohibitive for SCP production (FAO, 2010).

#### Summary

The knowledge of SCP production from the last century did not result in major industrial scale production of SCP after the 1980s due to high costs. Despite the nutritional value of SCP from each source looks promising, microalgae are the only source readily used as animal as well as human feed additive. Fungal biomass used for baking is discontinued today. The increasing resource limitations are expected to drive SCP production and improve the economic feasibility in the future. The application for wastewater treatment with simultaneous production of SCP in large scale is only applied for microalgae. Wastewater treatment with fungi is very limited whereby solid waste treatment seems economically prohibitive. PPB seems to offer another option, with effective wastewater treatment capacities, acceptable nutritional value and high yields but without current full-scale installation.

**Table 3** shows the theoretical biomass production based on nitrogen content of the wastewater assuming 30% utilization for soybean production, 25% for microalgae and 90% yield for PPBs. Comparing microalgae and PPB with agricultural plant growth, e.g., soybeans shows that nitrogen fertilizer can be used most effectively by PPBs. The overall yields for biomass production from microalgae and PPB in terms of nitrogen are far higher compared to plants because nitrogen is effectively assimilated. However, due to stripping of ammonia, the nitrogen losses in open HRAP are almost comparable to runoff and volatilisation in agriculture.

**Table 3:** Theoretical biomass production based general elemental composition of different sources and nitrogen content of various wastewaters.

Wastewater	Nitrogen (mg/L)	Soybean^∗^ (g biomass)	Microalgae (g biomass)^∗∗^	PPB (g biomass)^∗∗∗^
Weak domestic	20	0.1	0.1	0.2
Medium domestic	40	0.1	0.2	0.3
Strong domestic	85	0.3	0.3	0.6
Beef cattle feedlot	63	0.2	0.2	0.5
Dairy	185	0.6	0.7	1.4
Poultry feedlot	802	2.7	3.0	6.0
Swine feedlot	895	3.0	3.4	6.7
Paper mill	11	9.0	10.2	20.3
Winery	110	0.0	0.0	0.1

*Elemental compositions for; soybean meal (C:H_*1.8*_:O_*0.5*_:N_*0.2*_:P_*0.006*_) (Osborn, 1977); microalgae (C:O_*0.48*_:H_*1.83*_:N_*0.1*_:P_*0.01*_) (Chisti, 2007); PPB (C:H_*1.6*_:O_*0.4*_:N_*0.2*_:P_*0.02*_) (Ormerod, 1983) and ^∗^30% (Matassa et al., 2015), ^∗∗^25% (García et al., 2000), and ^∗∗∗^90% conversion of supplied nitrogen (Hülsen et al., 2015).*

### Recovery of Metals

Contamination of water sources by metals is of big concern. The origin of contamination is mostly related with anthropogenic activities including mining, metallurgical operations, burning fossil fuels, cement production, electroplating, leather tanning, and manufacturing activities as plastics, fertilizers, anticorrosive agents, Ni-Cd batteries, dyes, photovoltaic devices, pigments, or pesticides, among others (Nancharaiah et al., 2016). Some of the metals are finally disposed into DWWTP, the sources including partially treated industrial eﬄuents, disperse contamination points, runoff from roads as well as soil leachates from highly contaminated ponds and soils as uncontrolled landfills and mines. The metals are usually removed from DWW and accumulated in domestic sewage sludge, where more concentrated metals (>10 ppm) have been identified as Fe, Al, Ti, Zn, Cu, Sn, Mn, Cr, Mo, Ag, Ni, U, and V (Westerhoff et al., 2015), although this composition can substantially vary depends on the geomorphology and human activities.

Beside the inherent environmental and human concern of heavy metal contamination, there is an increasing opportunity to regain and recovery these resources for reusing and comply with the cradle-to-cradle concept for anthropogenic metals activities. Metals recovery by biological technologies have been studied for decades, but earlier studies dealt with biomining activities (use of microorganisms for extracting metals from mining sources) (Johnson, 2014) and heavy metals bioremediation (Mosa et al., 2016) rather than recovery from waste sources. The circular economy concept appeared in the mining and other metals-related industrial activities just a short while ago (Westerhoff et al., 2015). The accumulated experiences of biomining and bioremediation have been dedicated to the rising heavy metals recovery from wastewater paradigm and so many technologies under current analysis evolved from those concepts. **Table 4** shows a selected review of recent studies dealing with different biological technologies for metals recovery from waste and wastewater sources. Those technologies derived from biomining activities are linked to mobilization of metals (bioleaching by chelation, oxidation, and acidification), whereas technologies adapted from bioremediation techniques are more associated with immobilization of metals (bioprecipitation, bioreduction, biosorption, and bioaccumulation). There are some detailed reviews showing a big picture on metals recovery (Kikuchi and Tanaka, 2012; Johnson, 2014; Nancharaiah et al., 2016) but in this part the novelest and most impacting studies in recent dates are analyzed based on their link with the circular economy concept.

**Table 4:** Selected review of heavy metal recovery from waste and wastewater sources.

Metal/s	Source	Process	Mechanism/reaction^∗^	Microorganism involved	Recovery potential^∗∗^	Reference
Al, Mo, Ni, Va	Hydrotreating catalysts	Mesophilic bioleaching/chemical precipitation	M^0^ + 2Fe^3+^ → M^2+^ + 2Fe^2+^	Mixed culture	65 (Al), 87 (Mo), 52 (Ni), 65 (V)	Cibati et al., 2015
Cu, Ni, Zn, Pb, Ga, Sn	Printed circuit boards	Thermophilic bioleaching	M^0^ + 2Fe^3+^ → M^2+^ + 2Fe^2+^	Mixed culture (dominating *Leptospirillum, Acidithiobacillus, Sulfobacillus*	99 (Cu), 84 (Ni), 99 (Zn), 3 (Pb), 43 (Ga), 7 (Sn)	Guezennec et al., 2015
Cu	Cu-Ag ores	Mesophilic bioleaching	Glutamate → Glutamic acid + Cu_(s)_ → Cu^2+^_(aq)_	*Lysinibacillus sphaericus* JG-A12, *Bacillus* sp. JG-B12, *Bacillus* sp. JG-B5T	20–43	Kostudis et al., 2015
Fe	Synthetic wastewater	Chelation	Bioproduction of pyoverdin (Pyo, C_56_H_88_N_18_O_22_). Pyo + Fe(III)_(aq)_ → Complex Pyo-Fe(III)	*Pseudomonas fluorescens*	99	Renard et al., 2005
Pt, Pd, Rh	Spent automotive catalysts	Mesophilic bioleaching	Glycine → CN^-^ + NaOH → NaCN 2Pt + 8NaCN + O_2_ + 2H_2_O → 2Na_2_[Pt(CN)_4_] + 4NaOH	*Chromobacterium violaceum*	92.1 (Pt), 99.5 (Pd), 96.5 (Rh)	Shin et al., 2015
Ni, V, Mo	Decoked spent petroleum catalyst	Mesophilic bioleaching	S^0^ + 1.5O_2_ + H_2_O (b) → H_2_SO_4_ Mo_x_ + H_2_SO_4_ → MSO_4_ + H_2_O	*Acidithiobacillus thiooxidans*	79 (Ni), 90 (V), 88(Mo)	Srichandan et al., 2014
As(III), Fe (II)	Synthetic wastewater	Oxidation/Precipitation	Fe(II) + O_2_ → Fe(III)_(aq)_ → jarosite: [K, Na, NH_4_]Fe_3_(SO_4_)_2_(OH)_6_) As(III)(aq) + jarosite → As-Jarosite	Mixed culture (*Acidithiobacillus ferroxidans, Acidithiobacillus ferrivorans, Leptospirillum ferriphilum, Leptospirillum ferrooxidans*)	99.5	Ahoranta et al., 2016
Cu(II), Fe(II)	Acid mine drainage	Reduction/Precipitation	Lactate + SO_4_^2-^ → biomass + H_2_O + CO_2_ + S^2-^ Cu^2+^ + Fe^2+^ + 2S^2-^ → CuS + FeS	SRB Mixed culture	99 (Cu), 97 (Fe)	Chen et al., 2009
Fe, As, Cu, Cd, Zn, others	Acid mine drainage	Oxidation/Precipitation/Reduction/Precipitation	Fe(II) + O_2_ → Fe(III)_(aq)_ → schwertmannite: Fe_8_O_8_(OH)_6_ As(III)_(aq)_ + schwermannite → As-schwermannite Glycerol + SO_4_^2-^ + H^+^ → S^2-^ + CO_2_ + H_2_O S^2-^ + M^2+^ → MS	First stage: *Ferrovum mixofaciens*. Second stage: SRB Mixed culture	99.9 (Cd, Cu), 50 (Ni), 99.9 (Fe), 99.9 (As)	Hedrich and Johnson, 2014
Cd, Cu, Mn, Zn	Synthetic wastewater	Bioaccumulation	Direct bioaccumulation inside the cells	*Chlorella minutissima*	33.7 (Zn), 21.2 (Mn), 35.4 (Cd), 3.3 (Cu) mg/g	Yang et al., 2015
Pb	Lead-zinc mine tailings	Bioaccumulation/biomineralization	Pb^2^^+^ → Ca_2.5_Pb_7.5_(OH)_2_(PO_4_)_6_ (Pb-hydroxiapatite)	*Bacillus cereus*	226 mg/g	Chen et al., 2016
Au	Au-containing industrial wastewater	Biosorption	Direct biosorption onto EPS	*Cyanothece* sp., *Nostoc* sp., *Rhodopseudomonas palustris, Rhodobacter sphaeroides*	318 (*Cyanothece*), 64 (*Nostoc*), 80 (*R. palustris*), 45 (*R. sphaeroides*) mg/g	Colica et al., 2012
Fe	Barren head leaching eﬄuent	Oxidation/precipitation	Fe^2^^+^_(aq)_ + O_2_ +2H^+^ → Fe^3+^_(s)_ + 2H_2_O	Mixed culture (dominating *Leptospirillum ferriphilum* and *Ferromicrobium acidiphilum*)	5–40	Nurmi et al., 2009
As	Synthetic wastewater	Reduction/precipitation	As(V) + SO_4_^2-^ + Ethanol → As(III) + S^2-^ +Biomass → As_2_S_3_ + AsS	Anaerobic mixed culture	91.2	Rodriguez-Freire et al., 2016

*^∗^M, Metal; (aq), aqueous; (s), solid. ^∗∗^Data is in (%) except where specified.*

#### Heavy Metals

##### Mobilization

Dissolution of metals from rocks are natural processes where low pH usually creates a favorable environment for mobilization of metals into water bodies. The process is highly enhanced by microbial activity and some microorganisms known as extremophiles can in fact naturally convert a water body into a heavily contaminated site, as in the case of some rivers located close to Fe mining activities (Amils et al., 2011). This process involves three steps until complete mobilization of metals. Firstly, sulfide minerals exposed to oxygen are chemically oxidized to sulfate and Fe^2+^, creating an acidic environment. Under these conditions Fe^3+^ can further oxidize sulfide minerals via thiosulfate mechanism (in the case of pyrite -FeS_2_-, molybdenite -MoS_2_-, and tungstenite -WS_2_-) or via polysulfide mechanism (chalcopyrite -CuFeS_2_-, sphalerite -ZnS-, or galena -PbS-), mobilizing the metals. For the complete oxidation of these minerals it is necessary the chemolithotrophic sulfur-oxidizing microorganisms which can oxidize sulfur to sulfate (*Acidithiobacillus ferrooxidans, A. thiooxidans, Leptospirillum ferrooxidans*, and *L. ferriphilum*). However, without the action of these acidophiles that can also aerobically oxidize Fe^2+^ to Fe^3+^ and so regenerating the acidic-oxidizing environment, this process would follow much more slowly (Sánchez-Andrea et al., 2014).

These mechanisms have been traditionally used for industrial biomining with high level of success. Even, some minerals (Cu and Zn) are exclusively extracted by this process. The application of biomining to mobilize the metals contained in waste and wastewater sources has been therefore just a matter of time (Johnson, 2014). Recent applications of biomining have demonstrated the successfully recovery of metals from highly metallic industrial waste. Ni, V, and Mo were thus extracted from decoked spent petroleum catalyst (Srichandan et al., 2014), whereas mesophilic bioleaching has been used for extracting high-value metals from hydrotreating catalyst (Cibati et al., 2015). A clear opportunity to recover these kind of metals is the increasing quantity of landfilled electronic waste (e-waste), which commonly contains rare and precious metals as Au, Pt, Pd, Ag, and Rh (Pant et al., 2012). Biomining have been successfully applied to recover metals by co-processing of sulfidic mining wastes and metal-rich post-consumer e-wastes by biohydrometallurgy (Guezennec et al., 2015). However, extraction and recovery of metals from heterogeneous organic waste as domestic and industrial wastewater sludge is still a challenge and can also impact on decontamination of biological waste for subsequent direct use as valid and valuable resource (e.g., organic fertilizer or organic fuel) (Hennebel et al., 2015).

Other interesting processes for metal mobilization are biogenic cyanide production and metal chelation mediated by bioproducts. Cyanide is produced by oxidative descarboxylation of aminoacids like glycine, mediated by HCM synthase, in some species of *Pseudomonas* sp. and *Chromobacterium viollaceum*. Cyanide can be used for bioleaching of precious metals like those from the platinum group that are usually spent in automotive catalysts production (Shin et al., 2015). Some organics with chelating properties are glutamic acid, which has been previously used for leaching of Cu from Cu-Ag ores by *Bacillus* sp. (Kostudis et al., 2015), and pyroverdin production by *Pseudomonas fluorescens* immobilized on porous mesostructured silica that is capable of Fe(III) chelation (Renard et al., 2005).

##### Immobilization

Microorganisms are capable of creating an environment for immobilization of dissolved metals. Typical metals-removing microorganisms have been applied for decontamination of water and land spills, as well as for bioremediation of heavily metallic industrial wastewater (Mosa et al., 2016). However, recovery of metals immobilized onto biomass is a relatively new approach and only technologies involving recovery of dissolved metals in mine tailings have been commercialized so far (Huisman et al., 2006).

Biosorption processes are among the most studied non-destructive removal mechanisms for recalcitrant compounds. They are considered as non-biological mechanisms where the chemical (sorbate) – biomass (sorbent) interaction is only dependent on their mutual affinity (Vijayaraghavan and Yun, 2008). Most heavy metals can be passively adsorbed onto anionic functional groups present in external polysaccharides (EPS) and membrane lipids and proteins (Kikuchi and Tanaka, 2012), like peptidoglycan, phospholipids, lipopolysaccharides, teichuronic and teichoic acids and various proteins (Vijayaraghavan and Yun, 2008; Din et al., 2014). The sorption potential of heavy metals depends on pH, biomass loading, equilibrium time, initial metal ion concentration, temperature, and the method of the sorption process applied (Aryal and Liakopoulou-Kyriakides, 2015). Even precious metals like Au can bind onto EPS of bacteria (Colica et al., 2012). Since it is a surface process, most of the biovolume fraction is unoccupied and therefore heavy metals immobilization potential is limited. However, some bacteria and fungi are also capable of actively bioaccumulating the heavy metals following by some kind of chemical transformation inside the cells.

Bioaccumulation happens when bacteria make use of metals inside the cell for some metabolic or physiological benefit. For example, some bacteria are able to bioaccumulate Pb as hydroxyapatite nanocrystals [Ca_2.5_Pb_7.5_(OH)_2_(PO_4_)_6_] highly enhancing the phosphate and calcium bioavailability (Chen et al., 2016). In this line, bioaccumulation of Cd and Cu by microalgae *Chlorella minutissima* improves lipid production that can be furtherly used for energetic recovery of organics as biofuels (Yang et al., 2015). Metals accumulated within the cell can react with phosphate (*Pi*) giving insoluble metallic phosphates. These processes also happen in enhanced biological phosphorus removal (EBPR) systems and are due to the ability of some organisms to accumulate *Pi* as poly-P (phosphorus accumulating organisms, PAOs) (You et al., 2011). While poly-P is an energetic accumulative polymer potentially used as ATP reservoir, it may have a role in protecting PAOs from the toxicity of heavy metals. Bivalent and trivalent metals can interact with accumulated poly-P decreasing the inherent toxicity of these compounds to PAOs. These processes may be accounted for in the Fe-S-P nexus in aerobic and anaerobic wastewater technologies, linking to the expanded ADM1 model but in heavy metals-bearing eﬄuents (Flores-Alsina et al., 2016).

Metabolic reduction-oxidation processes also enhance the immobilization of metals in aquatic environments. According to its nature, the immobilization can be due to direct precipitation of metals as inorganic minerals or indirect precipitation due to the formation of anionic salts combined with biological alkalization of the medium. Some of these processes have been indeed included in the recent plant-wide aqueous phase chemistry module for overall wastewater treatment developed by Flores-Alsina et al. (2015) that accounts for Fe and Al speciation as well as other cationic components, which solubility can be greatly affected by the pH of the medium, the presence of anionic species and the effect of oxidation-reduction processes (Flores-Alsina et al., 2015).

The Fe-S-P nexus has a leading role in many redox/bioprecipitation processes. Sulfide generation by sulfate reducing bacteria (SRB) and *Pi* mobilization linked with Fe oxidation/reduction processes can be modulated for controlled metals immobilization. For example, biological oxidation of Fe(II) to Fe (III) can promote the recovery of Fe but also control the Fe concentration in bioleaching (Nurmi et al., 2009). Sulfate reduction and generation of sulfide can be used to selectively precipitate bivalent metals (Chen et al., 2009; Sánchez-Andrea et al., 2014). This process also explains why heavy metals toxicity is dramatically reduced when sulfide is produced during anaerobic culturing. Even during long-term metals leaching caused by corrosion and/or dissolution processes, the biogenic ferrous sulfide creates a protective barrier avoiding the metals to be in contact with highly sensitive methanogens (Gonzalez-Estrella et al., 2015, 2016). An interesting example of usage of the iron-sulfur cycle is the renmediation and recovery of arsenate by iron-sulfides and sulfates (Rodriguez-Freire et al., 2014, 2016). Other precipitative processes are indirectly related with microbial redox metabolism and are derived from biogenic alkalinity, as in the case of microalgae, fungi, and phototrophic bacteria technologies (Das et al., 2009; Ye et al., 2015).

#### Metallic Radionuclides

Radionuclides metals have unstable atomic structure with excess nuclear energy, which emits ionizing radiation that can affect genetic structure of most living beings, including microorganisms that can suffers acute radiation hormesis phenomenon (Kudryasheva and Rozhko, 2015). Even such properties are not an impediment to have biotechnological applications for radionuclides recovery from mineral rock or radioactive waste and wastewater (Francis, 2012). Most of the recovery technologies entail immobilization of the radioactive metals and recovery after de-structuring of biological waste.

Biosorption plays a key role on biological recovery of radionuclides. Uranium has been previously immobilized by using organisms such as *Rhodotorula glutinis* (Bai et al., 2014) and *Pseudomonas putida* (Choi et al., 2009) with efficiencies higher than 70%. Similarly, a simulated metal refinery wastewater containing high ammonium and rhodium concentrations was treated by nitrification-denitrification for N removal followed by Rh recovery by using different microbial adsorbents, achieving >50% recovery efficiency (Manipura and Burgess, 2008). Other microorganisms can actively increase their radionuclides sorption capacity. For example, non-sterilized active anaerobic bacteria, originally used for the treatment of pulp and paper wastewater, accumulated considerable amounts of plutonium, actinium and neptunium, in all the cases increasing their bioaccumulation capacity compared to sterilized biomass, strongly suggesting biologically mediated process (Sasaki et al., 2001). Also, some microbes can enhance chemisorption of radionuclides by promoting their interference with the Fe-S-P nexus. This is the case with *Citrobacter* sp. that can regulate its phosphate biochemical mechanisms to entrap Np and Pu and co-precipitate as metallic phosphates. The transuranic elements removal by this technology is via a hybrid of bioaccumulative and chemisorptive mechanisms (Macaskie and Basnakova, 1998).

Redox processes can also interfere with immobilization of radionuclides. Anionic Rh can be biologically reduced to Rh(III) by SRB at low pH inside the cell, process mediated by the enzyme hydrogenase. Subsequently, Rh is excreted and precipitated outside the cell. SRB can immobilized up to 66 mg/g of Rh using this mechanism (Ngwenya and Whiteley, 2006). Likewise, Pu can be immobilized by the indirect actions of microorganisms resulting in changes in Eh and its reduction from a higher to lower oxidation state, with the precipitation of Pu, its bioaccumulation by biomass, and bioprecipitation reactions (Francis, 2001).

#### Perspectives

Generally, most of metallic eﬄuents contain low concentrations of metals that convert the use of traditional chemical methods of recovery a chimera. Therefore, a step of pre-concentration of metals is essential but abiotic pre-concentration methods (e.g., nanofiltration, electrodialysis, or reverse osmosis) are costly technologies, especially for high flows as is the case with DWW and some industrial WW. Partitioning techniques involving microorganisms in novel DWW platforms (Verstraete et al., 2009; Batstone et al., 2015) can be combined with recent advances of metal recovery by immobilization (partition) followed by mobilization of metals from concentrated sludge (release). The metals can be subsequently recovered by chemical or even biological transformations into minerals, with the possibility of fractionation of the precipitation reactions by modulating the precipitant concentration as well as the pH of the medium. This multi-stage process would upgrade the current Partition-Release-Recover for metals recovery from wastewater, and is described in **Figure 1**.

### Bioproduction in Bioelectrochemical Systems

Balancing redox metabolism is one of the oldest challenges of living systems on earth and one that every living cell must master within its ecological niche. Nature has developed many different solutions for this challenge; however, each solution operates in tight constraints, as holding one’s breath will quickly demonstrate to every one of us.

These constraints apply not only in the environment, but also pose a major challenge in man-made systems, such as the fermentation plants in industrial biotechnology or treatment plants in environmental applications. In recent years a new technology has been developed to address this challenge: the bio-electrochemical systems (BES). In such a BES redox balance can be achieved without the oxidation of substrates or production of reduced by-products and instead electrons are donated to or gained from solid state electrodes and respective (a)biotic counter reactions. The BES is nowadays studied broadly as a system that allows microbes to conduct oxidative or reductive metabolism while using solid state electrodes as electron donors or acceptors (Harnisch et al., 2014; Tremblay and Zhang, 2015). The transfer of electrons can either be facilitated through a direct contact between the cell and the electrode or via soluble molecules that can exist in an oxidized and reduced state, so called mediators. These mediators diffuse through the system and donate and receive electrons at either the electrode surface or the bacteria and potentially could facility applications in suspension cultures. The successful scale-up of a BES will depend on maximizing electron transfer rates (Logan and Rabaey, 2012), but this will require a deeper understanding of the electron transfer mechanisms both at the anode and the cathode (Tremblay and Zhang, 2015) and will also require a reduction in the observed over-potential (Zhao et al., 2006; Freguia et al., 2008).

#### Microbial Fuel Cells

Bio-electrochemical systems were firstly considered as a green technique to produce electricity from waste water, a so called microbial fuel cell (Rabaey et al., 2004; Franks and Nevin, 2010; Janicek et al., 2014). In such systems the bacteria oxidize organic carbon to CO_2_ and use the anode as electron acceptor. BES technology experienced a dramatic development over the past decades with ever widening applications beyond being a power source (Rabaey and Rozendal, 2010; Logan and Rabaey, 2012; Tremblay and Zhang, 2015). Due to technical challenges (current densities, over-potential etc.) and due to the low value of electricity, bio-electricity still remains a niche application for remote deployment that lacks alternative power sources, for instance deep sea applications (Lovley and Nevin, 2011). In a recent development, the production of hydrogen or hydrogen peroxide have been considered as alternatives electricity (Foley et al., 2010) and these systems, termed microbial electrolysis cells, can be used in conjunction with wastewater treatment leading to recovery of phosphorous (Cusick and Logan, 2012) and methane production (Villano et al., 2013).

#### Microbial Electrosynthesis

Originally the term microbial electrosynthesis (MES) referred to the assimilation of CO_2_ by microbes using a cathode as the source of electrons, however, the term MES has constantly evolved and now considers more broadly microbial bioconversions powered by a BES and leading to production of organic chemicals.

On the side of the anode, electricity production dominated the focus so far, but in a recent study it has been shown that using a pure culture of a strict aerobe, breathing the anode allowed the high yield production of a chemical precursor for industrial antioxidant production (Lai et al., 2016). This is a key development, as it highlights the advantage of using a strict aerobe in such a system. The cells are unable to convert the substrate to undesired fermentation products and due to the constrained metabolism full oxidation to CO_2_ is low. The use of a pure culture also has the advantage that the field is opened up to metabolic engineering for rate enhancement of product diversification.

On the cathode side, many more works have been published that achieved production of chemicals. First demonstrated by the Lovley group (Nevin et al., 2010) pure cultures of *Sporomusa ovata* could produce acetate from CO_2_ and electricity. This could recently be extended to a mixed culture system (Jourdin et al., 2014), however, it is important to mention that acetate as a commercial product would struggle to be competitive and is now seen in this context as an interesting option for *in situ* COD production at waste water treatment plants. Methane can also be produced on a bio-cathode from CO_2_ (Villano et al., 2010a) but the commercial value of this remains to be seen.

#### Recovery of Nutrients and Metals

Bio-electrochemical systems technology can also be used to recover metals and nutrients. For instance as entioned above phosphorous can be recovered (Cusick and Logan, 2012) and also nitrogen removal and recovery either via electrodialysis (Thompson Brewster et al., 2016) or as ammonium bicarbonate from source-separated urine has been investigated (Tice and Kim, 2014). In addition, the BES also offers the possibility to recover metals fom wastewater, recently reviewed (Wang and Ren, 2014).

#### Future Perspectives

Apart from the mentioned challenges in maximizing the electron transfer rates and choosing a suitable mechanism, it is not trivial to decide which products could be made in a BES. There are three groups of targets that could in theory be pursued (i) the production of bulk chemicals such as biofuels, plastics or platform chemicals or (ii) the production of high value chemicals such as pharmaceutical precursors or hard to synthesize complex or chiral structures including antibiotics, pesticides, or herbicides and (iii) the production of inorganics such as metal complexes that can serve as fertilizer or a source of valuable metals. The problem here will be to find products with a real advantage over traditional production systems in the first group this means competing with the petrochemistry and highly efficient sugar based bioprocesses. In addition, downstream processing is a big challenge when producing chemicals or fuels from waste streams, especially when looking into chemical feedstocks, where the highest purity is needed. In the second group, the substrate cost is negligible, which makes it even harder to compete on a cost basis, but oxidative process on an anode for instance could avoid the formation of toxic oxygen adducts as a fermentation by-product (for instance epoxide formation in terpene production processes).

## Perspectives: Bioeconomy and Circular Economy

This review has focused on technologies which enable resource recovery. The drivers are clear, and are to translate technologies which would normally remove contaminants into a liquid or waste concentrate stream (or reactively dissipate them) into products that feed into the circular economy. This is not a massive shift from current practices, but instead of focusing the process on removal, it focuses on recovery. That is, multiple candidate technologies that would otherwise remove a contaminant are instead screened to those that allow the byproducts to be reused. As stated in section “Domestic Wastewater as Key Developmental Platform for Nutrient and Energy Recovery,” this can be through a complete reimaging of the treatment process, or slight modifications (for example using activated sludge in its granular form).

The three classes of product are carbon/energy, bulk nutrients (NPK), and metals and trace compounds. Recalcitrant high-value organics such as pharmaceuticals, pesticides etc. are not considered here. The main use for nutrients and metals is either as elemental inputs to the circular economy (not currently economic, but ultimately inevitable), and for use in carbon/energy products (generally economic if the carbon product is feasible).

A detailed economic analysis was done comparing conventional activated sludge with emerging technologies, including high-rate activated sludge, photo-membrane bioreactors, and mainline anaerobic treatment (Burgess et al., 2015). This used commodity products (e.g., electricity, nitrogen, and phosphorous). This showed that next generation technologies (assessed as a complete wastewater treatment platform) are generally capital cost neutral vs. existing technologies, and likely competitive particularly at larger scale. However, given the product value is relatively low (mainly electricity, bulk nutrients), there is not a strongly compelling economic driver to use next generation processes, given its higher risk. However, another consideration in the future is the value of carbon separate from the energy value in the wastewater. Until now, the focus has been on production of biofuels, particularly biomethane. This leaves bulk nutrients to be recovered (or more often, dissipated), and metals to be concentrated in sludge, mainly as metals sulfide. While this has resulted in resource utilization, ultimately, it dissipates the concentrated carbon to CO_2_ (albeit short-cycle CO_2_). We are now seeing a shift which recognizes the upvalued nature of carbon in wastewater, with its use in generating byproducts such as biopolymers, liquid biofuels, commodity chemicals, and possibly even animal feeds as SCP. The latter even offers a vector to transfer macro and micronutrients back into the manufacturing and agricultural product chain. However, it is still soon to predict the real impact of recycling these bioproducts into a global circular bioeconomy. In most of the cases, the technology readiness level (TRL) of the enabling technologies is still low (below TRL5), needing dedicated economic analyses like Life Cycle Assessment once pilot or demonstration plants are implemented.

## Author Contributions

DP managed and submitted the review and wrote the metals recovery chapter. DB co-managed the review and wrote the chapter dealing with domestic wastewater and the implications chapter. DP and DB also reviewed the paper carefully and are co-first authors. TH wrote the SCP chapter and reviewed the paper. SA wrote the biofuels chapter and reviewed the paper. MP wrote the PHA chapter and reviewed the paper. JK wrote the BES chapter and reviewed the paper.

## Conflict of Interest Statement

The authors declare that the research was conducted in the absence of any commercial or financial relationships that could be construed as a potential conflict of interest.

## Footnotes

1 http://bbi-europe.eu/
